# Chemical, Microbial Quality, and Risk Assessment due to Toxic Metal Contamination of Egusi (*Citrullus colocynthis L*.) Powder Sold in Selected Ghanaian Markets

**DOI:** 10.1155/2020/8862404

**Published:** 2020-12-11

**Authors:** Winifred Arthur, Jemima Ofori, Peter Addo, Nelson Amey, Nii Korley Kortei, Paa Toah Akonor

**Affiliations:** ^1^CSIR-Food Research Institute, P.O. Box M30, Accra, Ghana; ^2^University of Health and Allied Sciences, PMB, 31 Ho, Ghana

## Abstract

The present study was undertaken to investigate the physicochemical and microbiological qualities of melon seed powder sold in some open Ghanaian markets. Twenty-five samples of powder were collected randomly from each of four major markets and analyzed for moisture, pH, total ash, acid insoluble ash, and free fatty acids (FFA) using standard methods. The microbial population was determined using the pour plate method. Melon seed powder samples had a neutral pH (6.9-7.3) and contained significant amounts of ash (0.1-0.6%). Acid insoluble ash of powder from one market was high (0.6%) and possibly indicate contamination with siliceous earth material. FFA ranged between 4.1 and 11.6% for powder from the four markets. Levels of lead were higher (0.4-0.8 ppm) than other metals such as cadmium (0.02 ppm) and copper (0.3-0.6 ppm). HQ values >1 were recorded for Pb, implying a greater risk of toxicity to consumers. Counts for aerobic bacteria, fecal coliforms, and yeast and molds were in the range of 3.2-4.4, 1.6-4.0, 1.4-2.8, and 1.1-3.2 log CFU/g correspondingly for these organisms. *E. coli* was not detected in any of the melon seed powder samples analyzed. This study highlights the need for proper handling of melon seed during processing, storage, and distribution, to safeguard its quality for consumers.

## 1. Introduction

Melons are major food crops in sub-Saharan Africa and tropical regions. They belong to the *Citrullus* family which consists of a wide variety eaten as fruits, and their seeds are used in many dishes [1]. Major regions of cultivation are the Middle East and West Africa (Nigeria, Ghana, Togo, and Benin), where the crop is interplanted with maize, cassava, and yam [2]. The seeds of melons are commonly referred to as *egusi* in areas where they are eaten. This name generally applies to any of the several similar-looking seeds from cucurbit species (family *Cucurbitaceae*) such as cucumber, watermelons, musk melons, squash, and pumpkins [3].

Melon seeds are a good source of oil, protein, minerals, vitamins, and energy. The seeds contain 4.6 g carbohydrates, 0.6 g proteins, 0.6 g crude fiber, 33 mg vitamin C, 17 mg calcium, 16 mg phosphorus, and 230 mg potassium per 100 g edible seeds [4]. It is also an excellent source of minerals and essential amino acids [5], polyunsaturated fatty acids, and phospholipids and also contain significant amounts of tocopherols and phenolic compounds which are beneficial to humans (Mariod and Matthaus, 2008). While the oil may be extracted for cooking, frying, or soap-making, the whole seeds are a major soup ingredient and common component of daily meals [6]. The melon seed is used both as a condiment and thickener. The seeds are prepared for consumption by parching and pounding to free the seed coat from the kernel. This can be eaten either raw or cooked. Usually, it is milled into a powder and added to soups and (Oke, 2009). It may also be ground into a paste, molded into balls, and added as a meat substitute to daily meals [5]. Its importance is also reflected in the fact that it supplies significant amounts of protein and fat to diet and also improves flavor and palatability.

Melon seeds deteriorate quickly in storage due to fungal infection which may result in decreased nutritive value, color changes, increased peroxide value, reduced seed germination, and mycotoxin production [7, 8]. Therefore, if proper sorting of seeds is not carried out before milling, it may pose a health hazard to consumers. Besides, microbial attack, production, handling, and distribution practices may result in chemical changes that ultimately affect the quality and safety of the product. The accumulation of trace metals compromises the safety of food because they interfere with the proper function of the nervous system, kidneys, and other vital organs in the body. The presence of many of these elements has been reported in many foods. For instance arsenic, lead, nickel, copper, chromium, and cadmium have been detected in products of fruit and vegetable, cereals, root tubers, and meat [9, 10]. Although some of these (e.g., nickel, copper, and chromium) are essential elements, excessive amounts induce toxicity ([11].)

Processing, handling, and distribution of melon seed powder may affect its chemical and or microbial quality. This study was undertaken to investigate some chemical and microbiological properties of melon seed powder sold in major local Ghanaian markets.

## 2. Materials and Methods

The study was carried out using samples of melon seed powder obtained from four major markets in Accra, Ghana. The powders are produced by melon seed traders, who mill the seeds in market places where attrition mills are available. Melon seed powder was randomly purchased from 25 vendors in each market. A total of 100 powder samples were transported to the laboratory in clean polyethylene bags for analysis. Samples from each market were pooled into five groups and stored at 4 °C for chemical and microbial analyses. At each market, a semistructured questionnaire was used to gather sellers' demographic details and information on handling practices for melon seed or melon seed powder.

### 2.1. Chemical Composition of Melon Seed Powder

Moisture and total ash were, respectively, determined using approved methods 925.10 and 920.87 of the AOAC [12], while acid insoluble ash was determined following the method of Kirk and Sawyer [13].

### 2.2. pH Determination

The pH of the powders was determined following the method of AOAC [12], using a pH meter (Hanna, H14222) previously standardized with buffers 4 and 7.

### 2.3. Oil Extraction

Following the method of Opoku-Boahen et al. [14], oil was extracted from melon seed powder using a soxhlet extractor on a water bath, with petroleum ether as a solvent. The oil obtained was analyzed for free fatty acids.

### 2.4. Determination of Free Fatty Acids

Ethanol was mixed with 3 drops of 1% phenolphthalein solution and carefully neutralized with 0.1 M sodium hydroxide until a pink color was observed. Two gram of the extracted oil was dissolved in 40 mL of the mixed neutral solvent, heated to near boiling point, and titrated with 0.1 M NaOH shaking constantly until a pink color that persists for 15 s was obtained. FFA was expressed as mg KOH/g.

### 2.5. Determination of Trace Metal Contaminants

The Dry Ashing Method (AOAC 2005) was used for the Atomic Absorption Spectrometry (AAS) analysis. Flame Atomic Absorption Spectrophotometer (Buck Scientific, Inc. East Norwalk, USA) was used to read the absorbance values at the appropriate wavelength of the interested metal in the sample solution. The metal content of the sample was derived from a calibration graph made up of a minimum of three standards. To ensure the reliability of results, samples were handled carefully to avoid contamination. A recovery test for the analytical procedures was also carried out for the metals analyzed by spiking samples with aliquots of metal standards and reanalyzed for the selected metals. Acceptable recoveries of 95 ± 1%, 97 ± 1%, 95 ± 1%, 97 ± 1%, 94 ± 1%, and 95 ± 1% were obtained for As, Cd, Cu, Hg, and Pb, respectively.

### 2.6. Risk Assessment

#### 2.6.1. Determination of Estimated Daily Intake (EDI)

The estimated daily intake (EDI) is directly linked to the metal concentration, food consumption, and body weight. The following assumptions were made in this research to estimate the risk of heavy metals from *egusi* consumption at the extreme; the ingested dose was equal to the absorbed pollutant dose [15]; cooking did not affect the pollutants [16]; the average Ghanaians adult body weight was 75 kg [17]; the average daily consumption of *egusi* in Ghana is 30 g per day (average daily consumption in Nigeria according to [18]). People who obtain *egusi* from the Madina market will consume the same as *Agbo*, *Nima*, and *Dome* markets. Therefore, the EDI of toxic metals for adults was calculated as follows:

(1) $$\begin{array}{c} \text{EDI}=\frac{C\times C_{\text{cons}}}{{\text{B}}_{w}}, \end{array}$$

where *C* is the concentration of heavy metals in *egusi* (mg/kg wet weight), *C*_cons_ is the average daily consumption of *egusi* in the local area (30 g/day *B*_*w*_), and *B*_*w*_ represents the body weight (75 kg).

### 2.7. Determination of Hazard Quotient (HQ)

(2) $$\begin{array}{c} \text{HQ}=\frac{\text{EDI}}{\text{RfD}}, \end{array}$$

where HQ is the hazard quotient, and RfD is the reference dose (mg kg^–1^ day^–1^). HQ values of <1 signify unlikely adverse health effects, while HQ values >1 indicate a likely adverse health effect.

### 2.8. Determination of Microbial Population

Ten gram of sample was aseptically weighed into 90 mL of sterile salt peptone solution (XPS) containing 0.1% peptone and 0.8% sodium chloride with pH adjusted to 7.2 and homogenized in a Stomacher (model 4001, Seward Medical) for 30 s at normal speed. The 10^−1^ dilution obtained was vortex for about 2 min to ensure uniform mixing. One microliter of the 10^−1^ dilution was pipette into 9 mL of sterile salt peptone water to obtain 10^−2^ dilution. This procedure was repeated for 10^−3^, 10^−4^, 10^−5^, and 10^−6^ dilutions. An aliquot (1 mL) of each dilution was inoculated into sterile plates and the appropriate media added for enumeration and isolation using the pour plate method. Total plate count was enumerated using plate count agar medium (OXOID Chemicals Ltd, Basingstoke, UK) and incubated at 30°C for 72 h [19]. Coliform bacteria were enumerated on Tryptone Soya Agar medium (OXOID Chemicals Ltd, Basingstoke, UK) and incubated at 37°C for 24 h [20]. *E. coli* bacteria were enumerated on Trypsin Soya agar medium (OXOID Chemicals Ltd., Basingstoke, UK) and incubated at 44°C for 24 h [21]. Yeast and mold were enumerated on Dichloran Rose Bengal Chloramphenicol (DRBC) medium (OXOID Chemicals Ltd., Basingstoke, UK), to which 1% chloramphenicol in absolute ethanol was added as a supplement to suppress bacteria growth and incubated at 25°C for 3-5 days [22].

### 2.9. Statistical Analysis

Statistical analyses on the data were analyzed using one-way ANOVA. Where appropriate, the difference between means was separated using Duncan's Multiple Range Test, at a 95% confidence interval (SPSS 17.0.1, SPSS Inc.). Demographic data were presented as percentages.

## 3. Results and Discussion

### 3.1. Demography and Storage Practices of Vendors

Background studies from the various markets revealed that only women were involved in selling *egusi* powder and or seeds. These sellers had attained up to basic level education (Table 1). More than half of the vendors had been in the business between 6 and 10 years while a few had more than 10 years' experience.

**Figure 1:**
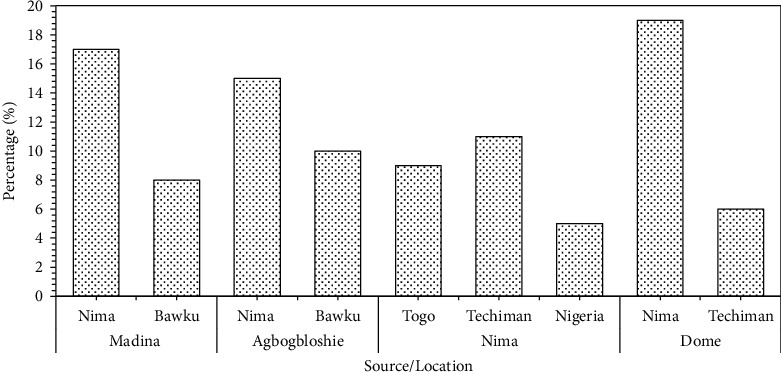
Sources of melon seed supply.

The results showed that Bawku, Techiman, Togo, and Nigeria are the main sources of melon seeds for these major markets (Figure 1). The majority of sellers from Madina, Agbogbloshie, and Dome markets obtained their melon seeds from the Nima market and other locations such as Bawku and Techiman. It is reasonable, therefore, to suggest that any form of contamination that may occur in the product at the Nima Market is likely to be carried over to the other markets. Traders in Nima trace the source of their supply to Techiman (in Brong Ahafo Region of Ghana) and neighboring West African countries Togo and Nigeria. This emphasizes the similarities in diets in the people of West Africa and the free movement of food crops across the borders of these countries. Once the vendors obtain the *egusi* seeds, they process some portion into powder and sell the remaining as whole seeds. The vendors indicated that they clean and sort the seeds before milling into powder using commercial disc attrition mills located on the markets. These commercial mills were observed to be housed predominantly in wooden structures fitted with windows with no mesh to keep away flies and other insects.

**Figure 2:**
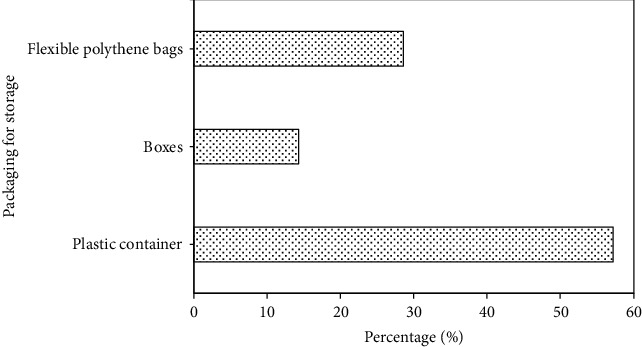
Packaging material for bulk storage of egusi powder by vendors.

Most of the sellers indicated that a batch of *egusi* powder lasts a maximum of seven days, but this heavily depends on patronage. The powder, according to the women, is usually kept in HDPE bowls/buckets from which measured portions are scooped and sold to consumers (Figure 2). The powder is sold in unbranded, clear, and flexible polyethylene or polypropylene bags and secured with a knot. This practice is likely to increase the risk of rancidity occurring in the powder whiles in storage since it is often exposed to air and moisture.

### 3.2. Physicochemical Properties of Melon Seed Powder

Table 2 shows the results of physicochemical analysis of *egusi* powder within the various markets. The chemical properties of melon seed powder from the four markets showed slight variations in the levels of the various indices assessed.

Moisture is a very important parameter when considering the quality of food powders and their acceptability. It affects their shelf life and is normally the key determinant of microbial attack during storage [23–25]. Generally, the moisture content of various samples of melon seed powder from the four markets ranged between 4.9 and 5.7% (Table 2). The moisture content of the powders may be low enough to prevent caking and reduce microbial proliferation and chemical reactions that may occur during storage. Moisture levels obtained in the present study were consistent with the findings of Riaz et al. [26] and Sadou et al. [27] who correspondingly reported percentage moisture of 6.4 and 4.9% for *Citrullus colocynthis* seeds. Other researchers [28, 29], however, obtained slightly lower moisture levels (4.3 and 4.4%) for the same species of *Citrullus colocynthis* seeds.

The pH of the powders was neutral and ranged between 6.9 and 7.3, with significant differences observed between samples from the Madina market and the others (Table 2). These results exclude the likelihood of fermentation in the melon seed powder sampled from these markets. pH of the powders may, however, be favorable for the growth of many microorganisms, especially bacteria. Microorganisms have different moisture content and pH optima for their growth. Most bacteria grow best at about pH 7 and poorly at pH below 4 [25].

Total ash, which reflects the mineral content of the product, varied slightly between powder samples obtained from the different markets. Indeed, ANOVA revealed that powder sampled from the Dome market had a significantly (*p* < 0.05) higher amount of ash, compared to the other markets. The results suggest different levels of mineral elements in the oilseed powders, an observation that may be ascribed to environmental conditions under which they were cultivated. Ash values obtained here were higher than ash (2.9%) reported by Milovanovic and Picuric-Jovanovic (2005), but lower than the range (4.5-5.1%) reported by Petkova and Antova (2015). Discrepancies in the results from these may be attributed to differences in melon varieties from which seeds were obtained and environmental conditions under which each was cultivated.

Melon seed powder procured from the Dome market had the highest acid insoluble ash or 0.6 g/100 g (Table 2). Samples from the other markets were markedly lower in acid insoluble ash, with values ranging between 0.02 and 0.06 g/100 g. Acid insoluble ash is an indicator of contamination with sand and other siliceous earth material. The value for samples analyzed from the Dome market may indicate some level of contamination with siliceous earth material. This may be traced to inefficient sorting practices that leave particles of sand and stones in the melon seeds during processing.

Free fatty acids are very important in determining the use of oil for industrial or culinary purposes. Their presence in oil may result from unsatisfactory processing, lipase activity, or hydrolysis [30], which may occur during storage. FFA content of melon seed powder ranged from 4.1 to 11.6 mg KOH/g with samples from Nima recording the highest value (11.61 mg KOH/g) and Dome recording the lowest (4.09 mg KOH/g). The high FFA content of the melon seed powder samples may also be due to the presence of active lipase in the seeds, which upon milling hydrolyzes triglycerides into free fatty acids, diglycerides, and monoglyceride. Mode of storage for bulk melon seed powder may also have contributed to the results obtained since traders keep the powder in plastics bowls/buckets (Figure 2), often exposed to air, which may facilitate rancidity. Levels of FFA observed in the present study were higher than Obasi et al. [29] and Riaz et al. [26] who, respectively, reported FFA of 3.09 and 1.09 mg KOH/g in *C. colocynthis*.

### 3.3. Trace Metal Contamination of Melon Seed Powder

**Figure 3:**
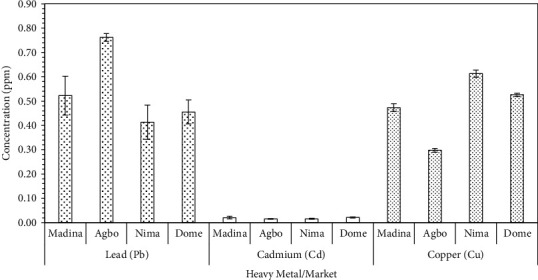
Levels of trace metal contaminants in melon seed powder from four markets.

Through food consumption, the body could easily become contaminated with heavy metals, since these elements are either absorbed from the soil or released during food processing. Their toxicity may affect mental and nervous systems and other vital organs [31, 32]. In this study, arsenic (As) and mercury (Hg) were not detected in any of the melon seed powder sampled from the various markets. The remaining three metals, namely, lead (Pb), cadmium (Cd), and copper (Cu) were detected (Figure 3).

Among the metal detected, levels of Cd were the lowest and ranged between 0.015 and 0.021 ppm, with no significant differences (*p* > 0.05) between levels observed for the various markets. This concentration is below the 0.1 ppm suggested for legumes and pulses or the provisional tolerable monthly intake (PTMI) of 25 *μ*g/kg body weight (JECFA, 2010). Chary et al. [33] explained that because Cd is highly mobile and poorly adsorbed in soils, it is easily absorbed by plants. However, JECFA (2010) indicates that the daily ingestion of Cd in food has an almost negligible effect on overall exposure because of the long half-life of this metal. The concentration of Pb and Cu was 0.4–0.8 ppm and 0.3–0.6 ppm correspondingly for the two metals. Pb may occur through absorption from the soil or contamination through processing equipment. Unlike Cd, Pb accumulation in plants is slower and occurs when soils contain high concentrations because it is tightly bound to soil colloids [33]. Significant variation (*p* ≤ 0.05) in levels of these two metals was recorded for melon seed powder obtained from the four markets. Melon seed powder obtained from Agbogbloshie and Nima markets, respectively, had the highest concentration of Pb and Cu. This observation suggests that the milling of seeds into powder may have contributed to the levels of these elements in the powder. Indeed, previous studies [34–37] reported that the use of a disc attrition mill contributes to heavy metal accumulation in food. Kwofie et al. [36] explained that the grinding discs in these attrition mills are fabricated by local artisans using unalloyed cast iron. This material is not resistant to wear and corrosion and therefore its usage may result in the release of some of the metals into food. An analysis of discs in previous studies revealed considerable levels of Pb and other heavy metals, and these gradually wear off into food at a high rate [34]. The level of Pb in the powders was higher than the tolerable limits of 0.3 ppm (for Pb in vegetables) set by the FAO/WHO joint committee [38].

#### 3.3.1. Risk Assessment due to Heavy Metal Contamination

The international guidelines for each heavy metal and their calculated Hazard Quotient (HQ) for samples from the markets are are shown in Table 3. Egusi powder obtained from the various locations had different risk potential. The estimated daily intake of the toxic metals analyzed ranged from 0.055-0.1, 1.33 × 10^−3^ − 2.67 × 10^−3^, and 0.04-0.081 mg/kg Bw/day, respectively, for Pb, Cd, and Cu. Calculated Hazard Quotients (H.Q) were also in ranges of 15.7-28.85, 1.33-2.67, and 1.00-1.73 again for Pb, Cd, and Cu (Table 3), suggesting a potential risk of heavy metal toxicity since they were >1 [39].

Variations in the EDI and HQ values for the egusi samples from the different locations could be largely attributed to the different alloy compositions of the attrition mills used for milling. There is also a possibility of bioaccumulation of these toxic metals by plant uptake via leached toxic elements in the ecosystem [40, 41].

Some researchers [42, 43] explained that the possible sources of toxic metals in the soil environment and agriculture are atmospheric deposition, livestock manure, irrigation with wastewater or polluted water, metallopesticides or herbicides, phosphate-based fertilizers, and sewage sludge-based amendments. Furthermore, natural sources, conventional/emerging anthropogenic contaminants pose major human health risks through the dietary intake of food crops contaminated by root transfer from soil to plant tissues or direct atmospheric deposition onto plant surfaces (Samsøe-Petersen et al., 2002; Zhuang et al., 2009). Particulate matter (PM) emitted by industries and vehicles ultimately accumulates in soil and end up in the food chain [40, 41].

### 3.4. Microbial Population in Melon Seed Powder

The microbial population of food is an important indicator of food safety and quality. High microbial count in food poses a danger to the health of consumers, and their presence may also contribute to decomposition and depletion of nutrients. The pH of oilseeds is near neutral, and this makes them susceptible to a diversity of microorganisms [25]. Results of the microbial assessment of melon seed powder from the four markets (Table 4) showed the population of aerobic bacteria, coliforms, yeast, and molds. No *E. coli* was recorded in any of the powder samples obtained from these markets.

Coliform count, TPC, and *E. coli* is an indicator of the hygiene status of food. TPC and coliform count also give information regarding shelf life and organoleptic changes during storage of the food stuff [24]. Aerobic plate count, coliforms, and the other microorganisms were higher in samples obtained from Agbogbloshie and Madina markets. The heaviest load of fecal bacterial (4 log CFU/g) was found in samples from the Agbogbloshie market. Generally, melon seed powder from the Dome market had the lowest microbial population. Samples from this market were, at least, one log unit lower in all the indicator organisms, compared to the other markets. TPC, yeast, and mold counts were higher than values reported by Ibeanu et al. [44] for seed flour mix, but less than 10^5^, 10^3^ specified correspondingly for yeast and molds in composite flours [45]. *E. coli* was also consistent with this same standard, but total coliform counts were higher.

The high coliform and TPC counts from this study may be ascribed to improper handling of melon seed powder and poor sanitary practices, which indicate inefficient process controls. Milling methods, residue build-up in milling machines, and storage practices may constitute a significant source of microbiological contamination which may negatively affect the safety and health of the consumer. Evidently (indicated by the survey results), the vendors store the powders in flexible pouches, wooden or plastic boxes, and plastic bowls/buckets. This practice may easily expose the food product to microbial contamination and adversely compromise its safety.

## 4. Conclusion

The study showed the presence of fecal coliform, yeast, and mold in amounts that may pose a health threat to consumers. The highest coliform count of 4.0 log_10_ CFU/g was recorded in one of the markets. The moisture content (4.8-5.7 g/100 g) of powder from the various markets was low enough to ensure product stability in storage. The powders contained significant amounts of ash, which is important in human nutrition. Acid insoluble ash of samples from one market was high (0.6%) and suggestive of contamination with sand or earth materials. The neutral pH, however, does not present an efficient restriction to microbial attack and therefore melon seed powder must be processed and handled carefully. This would minimize the possibility of contamination and ensure that the product is of good quality and is safe for consumption. Melon seeds investigated contained marked levels of heavy metals which poses a potential risk to consumers since their HQ values were greater than unity (>1).

## Figures and Tables

**Table 1:** Demographic information of respondents.

Variables	Groups	Percent (%)
Age (years)	20-29	41
	30-39	36
	40-49	14
	50 or older	9

Educational status	None	35
	Basic	60
	Secondary/vocational	5

Experience (years)	1–5	32
	6–10	53
	>10	15

**Table 2:** Chemical properties of melon seed powder from four Ghanaian markets.

Sample	Moisture (%)	pH	Total ash (%)	Acid insoluble ash (%)	Free fatty acids (mg KOH/g)
Madina	5.67 ± 0.21^b^	7.33 ± 0.09^b^	3.51 ± 0.09^a^	0.06 ± 0.04^a^	5.86 ± 0.93^b^
Agbogbloshie	5.35 ± 0.12^b^	6.92 ± 0.07^a^	3.47 ± 0.14^a^	0.05 ± 0.02^a^	4.37 ± 0.40^a^
Nima	5.46 ± 0.11^b^	6.93 ± 0.08^a^	3.45 ± 0.11^a^	0.02 ± 0.01^a^	11.61 ± 0.97^c^
Dome	4.88 ± 0.15^a^	6.97 ± 0.02^a^	4.04 ± 0.14^b^	0.57 ± 0.05^b^	4.09 ± 1.21^ab^

Mean within a column with different superscript are significantly different (*p* < 0.05).

**Table 3:** The estimated daily intake (EDI) and Hazard Quotient (H.Q) of toxic metals in egusi from the various locations.

Trace element	Location	USEPA RfD	Concentration (ppm)	EDI (mg/kg Bw/day)	HQ
Pb	Madina	3.5 × 10^−3^	0.53	7.07 × 10^−2^	20.20
	Agbogbloshie	3.5 × 10^−3^	0.76	1.01 × 10^−1^	28.85
	Nima	3.5 × 10^−3^	0.41	5.50 × 10^−2^	15.70
	Dome	3.5 × 10^−3^	0.45	6.00 × 10^−2^	17.14

Cd	Madina	1.0 × 10^−3^	0.02	2.67 × 10^−3^	2.67
	Agbogbloshie	1.0 × 10^−3^	0.01	1.33 × 10^−3^	1.33
	Nima	1.0 × 10^−3^	0.01	1.33 × 10^−3^	1.33
	Dome	1.0 × 10^−3^	0.02	2.67 × 10^−3^	2.67

Cu	Madina	4.0 × 10^−2^	0.47	6.30 × 10^−2^	1.58
	Agbogbloshie	4.0 × 10^−2^	0.30	4.00 × 10^−2^	1.00
	Nima	4.0 × 10^−2^	0.61	8.10 × 10^−2^	2.03
	Dome	4.0 × 10^−2^	0.52	0.069	1.73

Average daily consumption = 30 g; the average weight of Ghanaian adult = 75 kg. RfD: Reference Dose (USEPA,2012; Chauhan and Chauhan, 2014).

**Table 4:** Microbial population (log_10_ CFU/g) in melon seed powder from four markets.

Sample	TPC	Coliforms	*E. coli*	Yeast	Molds
Madina	4.3 ± 0.3	3.1 ± 0.3	0	2.8 ± 2.3	1.1 ± 0.5
Agbogbloshie	4.4 ± 1.0	4.0 ± 0.4	0	2.1 ± 0.5	3.2 ± 1.3
Nima	3.9 ± 2.3	2.7 ± 2.2	0	2.5 ± 1.5	2.9 ± 3.1
Dome	3.2 ± 0.1	1.6 ± 0.6	0	1.4 ± 0.2	2.2 ± 0.2

TPC: Total Place Count.

## Data Availability

All the data generated from this study have been presented in the manuscript.
